# Multi-Scenario Simulation of Green Space Landscape Pattern in Harbin City Based on FLUS Model

**DOI:** 10.3390/ijerph20054286

**Published:** 2023-02-28

**Authors:** Xue Li, Wen Li, Yu Gao

**Affiliations:** 1 College of Landscape Architecture, Northeast Forestry University, Harbin 150040, China; 2 Heilongjiang Forest Protection Research Institute, Harbin 150040, China

**Keywords:** green space, landscape pattern, multi-scenario simulation, FLUS model

## Abstract

In this study, the change in green space in different scenarios and the index characteristics of landscape patterns were analyzed and were conducive to providing the decision basis for future green space planning in Harbin, a city in Northeast China. The FLUS model was used to predict the layout of green space, and the prediction results were analyzed and evaluated using the landscape index method. Combined with the MOP model and LINGO12.0, the objective function of economic benefit and ecological benefit was established to maximize the comprehensive benefit. As revealed by the outcome, from 2010 to 2020, the fragmentation degree of cultivated land, forest, and grassland decreased, and the overall landscape level tended to be diversified and uniform. In the status quo scenario, the cultivated land and the forest land were increased, whereas the water area and the wetland changed little, and its overall benefit was the lowest. The forest was increased by 137.46 km² in the ecological protection scenario, the largest among the three scenarios, and the overall water quality improved. In the economic development scenario, the cultivated land tended to expand rapidly, the connectivity was increased, and the area of forest was decreased by 69.19 km², and its comprehensive benefit is lower than that under the scenario of ecological protection. The sustainable development scenario achieved the most significant economic and ecological benefits, with a total income of CNY 435,860.88 million. Therefore, the future green space pattern should limit the expansion of cultivated land, maintain the spatial pattern of woodland and wetland, and enhance the protection of water area. In this study, Harbin green space was studied from different scenario perspectives, combined with landscape pattern index and multi-objective planning, which is of great significance for Harbin green space planning decisions in the future and improving comprehensive benefits.

## 1. Introduction

Natural disasters happening more frequently and humans engaging in more activities have caused a variety of environmental issues (e.g., global warming, local climate change, air pollution, energy shortage), expedited the evolution of landscape structure, and hindered the long-term sustainability of both nature and humanity [1]. Green space, a vital part of the urban ecosystem [2], is capable of sequestering carbon and releasing oxygen [3], reducing the greenhouse effect [4], and carrying out rainwater regulation and storage [5,6]. It also takes on a certain significance in alleviating heat islands and can increase property values. Zhao et al. [7,8] studied the relationship between land use/land cover (LULC) type and surface temperature (LST) in Shenyang City. It was discovered that different types of LULC had dramatically different temperature distributions and that greenery and water had a considerable impact on the urban heat island (UHI) effect. In addition, the urban surface heat island intensity (SUHII) varies significantly in different months, and the applicability of the local climate zone (LCZ) scheme to land surface temperature (LST) differentiation also varies with month. Zhang et al. [9] found that the growth of green roof implementation (GRI) can be potentially motivated by property value enhancement and employment improvement, and the lack of government policies, unsound technical level, unsound economic benefit assessment, and weak personal will restrict the development of GRI. According to the classification system of green space in previous studies [10,11,12], green space includes cultivated land, forest, grassland, wetland, and water area, whereas non-green space comprises artificial surfaces and bare land. The research objects cover cultivated land, forest, grassland, wetland, and water area in Harbin.

Urban landscape structure and ecological service function are dependent on the green space landscape pattern, affecting how much it functions [13,14,15]. Landscape pattern analysis can determine the law, which takes on a significance for guiding the future in the chaotic landscape unit [16]. The evolution characteristics of landscape patterns were analyzed, the spatiotemporal change rule of landscape patterns was revealed, and the structural characteristics of different landscapes were compared using the quantitative analysis method, the landscape pattern index method, and the moving window method. Dadashpoor et al. [17] analyzed land use and land cover change (LUCC), urbanization, and landscape pattern using spatial indicators and the landscape expansion index (LEI). They used ordinary least squares (OLS) and geographically weighted regression (GWR) to analyze the relationships between the three changes. Su et al. [18] qualitatively examined the effects of urbanization on an eco-regional scale while analyzing the changes in landscape patterns and ecosystem service value in the Hangjia-Hu region. Lv et al. [19] examined the spatial and temporal changes in landscape patterns in the Dongjiang River Basin from 1990 to 2016 using a transfer matrix, moving window approach, and landscape pattern index.

The simulation of a future landscape pattern change in terms of LULC has been the subject of an increasing number of studies. Future scenarios can be built to model and study LUCC, the sources of change can be examined [20], and a reference base can be offered for regional planning to help decision-makers make wise decisions about land use planning [21,22]. Landscape dynamic attitude can quantitatively describe the speed of regional land use change, which is convenient for the comparison of regional differences and the prediction of the future trend of land use change [23,24]. Studies of future LULC should consider the growth of the regional economy besides environmental preservation. A wide range of land use simulation models (e.g., cellular automata (CA) [25], the conversion of land use and its effects modeling framework (CLUE) model [26,27], and the patch-generating land use simulation (PLUS) model [28]) have been developed to more effectively balance the conflict between ecological protection and economic needs to forecast future land use change. Multi-scenario simulation is essential for future planning. Our LUCC projections in this study are based on the future land use simulation (FLUS) model [29], a method that interactively combines bottom-up CA models with top-down system dynamics (SD) models. Since most of the other models cannot consider the effects of quantity and space-time on land use, this model considers the mutual effects of a wide variety of land-type conversion processes, eliminating the limitations of previous studies in obtaining land-type conversion rules by linear regression method [30], and studies show that the simulation accuracy of the FLUS model is higher [31]. Huang et al. [32] investigated the Shenyang urban growth boundary development model in a wide range of development scenarios using the CLUE-S model through an evaluation of the feasibility of the land for development. Zhang et al. [33] studied land use change in the Aksu region in multiple scenarios using the MOP-PLUS model. Fu et al. [34] evaluated the three types of space in Panlong District based on information entropy and dominance, in conjunction with the ecological protection red line, the permanent basic cultivated land protection red line, and the FLUS model.

The maximization of land use benefits through multi-objective decision making has become a prevalent area of research in land use planning [35]. Due to the rationale for creating a scenario, multi-scenario simulation frequently fails to produce the ideal development scenario. The Pareto optimal solution set, aiming to resolve the multi-objective scenario without abandoning any goals and maximizing the advantages of other goals, was proposed by economist Vilfredo Pareto [36]. Stewart et al. [37] optimized the spatial distribution of land resources and the ideal point approach using the evolutionary algorithm to address the multi-objective decision-making problem. Zhou et al. [38] took future land use parameters as fuzzy variables to optimize the county land use structure under multi-objective conditions. Zhao et al. [39] linked the MOP and FLUS model and optimized the production–life–ecological space’s spatial layout to maximize the spatially comprehensive advantages of the production–life–ecological space. In addition, these researchers used the landscape pattern index to examine and evaluate the optimization results.

The relevant studies [40,41,42,43,44] have mostly concentrated on multi-scenario simulations for areas of South China and North China, such as Shenzhen City [45], Chongqing City [46], Tibet [47], and the middle and lower reaches of the Yangtze River [48,49,50], with less research on green space in Northeast China. Cai et al. [50] analyzed the changes in landscape pattern and ESV in coastal areas of Fujian over the past 20 years using a patch generation land use simulation model, landscape pattern index, and ecological service value estimation method. Yang et al. [51] forecasted the land use change and landscape pattern of Zhangjiajie in 2030 using GeoSOS-FLUS software and proposed an optimization strategy for the future development of Zhangjiajie City. Nie et al. [52] built a land use simulation model using the PLUS model with coupling constraints of an ecological security model (ESP) and a multi-scenario (MS) model to develop land management policies for Anji County. Park et al. [53] showed the different performance of landscape indicators under different urbanization conditions, and which type of landscape was most likely to be sensitive to future urbanization process. Troupin et al. [54] simulated two scenarios of unregulated and regulated development in the Mediterranean region of Israel for the next 20 years and compared the two scenarios under different development rates.

In recent years, scholars at home and abroad have focused on the evolutionary driving mechanism [55,56], dynamic evolution analysis [57], and the cooling effect of green space [58,59]. The amounts of green patches from remote sensing data were primarily used in the investigation of the dynamic evolution of the green spatial pattern [12,60]. The study techniques mainly concentrated on large data analysis [61], landscape index analysis [62], spatial correlation analysis [63], and remote sensing technology [64]. The majority of current studies are qualitative studies, most of which have undertaken extensive studies and produced conclusive findings on the dynamic evolution traits and driving mechanisms of green spatial landscape patterns and ecological function effects in the past time and space. However, only a small number of studies have been carried out to forecast the future green spatial pattern with diverse scenarios, and only a limited number of studies have quantitatively examined the optimization of green spatial structure and its comprehensive advantages.

Combining the aforementioned applied studies with associated scientific theories reveals that most scholars are only capable of analyzing and forecasting changes in land use areas. They do not, however, provide multi-scenario forecasts or benefit assessments for patterns of urban green space, and their focus is also skewed toward southern cities at the expense of Northeast China. However, the northeast is the key to high-quality development in the new era [65]. Green space is the ecological base of a city, and the study of the spatiotemporal dynamic evolution of urban green space patterns is helpful for us to have a more intuitive understanding of the green space situated in the study area. The scenario simulation prediction of green space can assist in analyzing the cause-and-effect relationship of its changes, expanding the knowledge and experience of decision-makers in guiding rational land use and planning, and promoting the positive evolution of urban green space landscape patterns [11]. A reasonable green spatial pattern takes on a critical significance in optimizing the urban ecological environment and improving urban biodiversity, while the direction of urban development planning directly affects the urban green spatial pattern [66]. In addition, based on the objective conditions and combined with the current situation of the study area, this study constructed the objective function of economic benefit and ecological benefit and realized the optimization of the quantity structure of green space.

Based on the above background, this study chooses the green space of Harbin as the research object. Harbin, the capital of Heilongjiang Province, is an important city in Northeast China. It is undergoing rapid economic development and facing increasingly acute ecological and environmental problems. The emphasis on the environment is progressively deepening, and the pattern of green space in this region is constantly changing [67]. According to the Harbin City people’s government website (http://www.harbin.gov.cn/col/col394/index.html accessed on 1 January 2023), district national spatial planning and related policies are temporarily not issued. Accordingly, the temporal and spatial evolution characteristics of green space spatial patterns in the study area were explored using the landscape dynamic attitude model, the FLUS model, the MOP model, and the landscape pattern index. This investigation was conducted to clarify the intensity and trend of green space expansion in Harbin City from 2010 to 2020, as well as the trend of the landscape pattern of green space under different scenarios, and the land use structure under the optimal sustainable development scenario. The ideas elucidated are as follows: First, the landscape dynamic degrees of the respective components of Harbin’s green space were investigated from 2010 and 2020 (i.e., cultivated land, forest, grassland, wetland, and water area). Second, the evolution characteristics of green space coverage in status quo development scenarios, ecological protection scenarios, and economic development scenarios in 2030 were predicted using the FLUS model. Third, the evolution traits and degree of green space fragmentation at the class level and landscape level were analyzed using the method of the landscape pattern index in different scenarios. Lastly, the MOP model and LINGO 12.0 were integrated to determine the green space coverage of the optimal sustainable development scenarios. On that basis, more insights can be gained into the land use of Harbin’s green spaces, which takes on a great significance in optimizing the spatial distribution of the above-described areas, implementing sustainable urban growth in Harbin, and providing the rationale for future green space planning in Harbin.

## 2. Materials and Methods

### 2.1. Research Area

**Figure 1:**
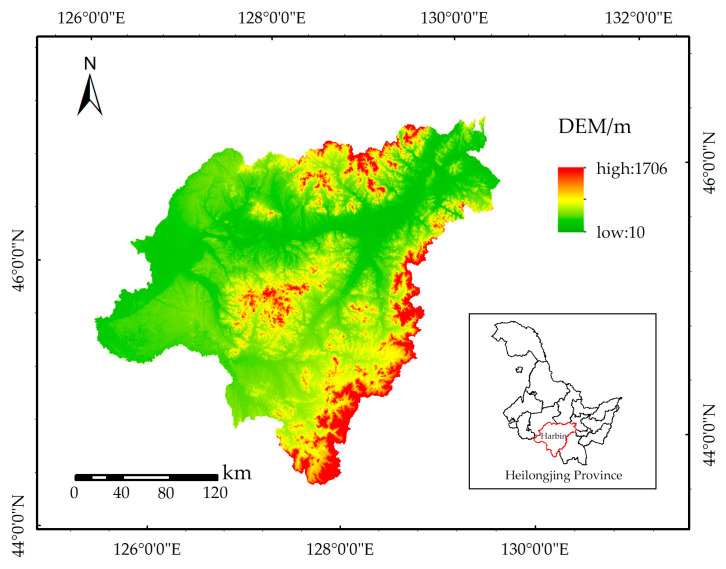
Geographical location and DEM map of the study region in 2020.

Harbin (125°42′~130°10′ E, 44°04′~46°40′ N) is located in the southern part of Heilongjiang Province, with a total area of 53,076.43 km² (Figure 1). Harbin is characterized by four distinct seasons (e.g., a long winter and a brief summer) and is located in a temperate continental monsoon climatic zone. The annual average temperature range reaches 4.60 °C, with an annual average precipitation of 827.50 mm.

Harbin was taken as the case study area for the following two reasons: (1) Harbin serves as Northeast China’s economic, political, and cultural hub. It is where the Ha-Da-Qi Industrial Corridor begins and where the international aviation traffic corridor is centered. It also has a long history and cultural heritage. The examination of the green space landscape pattern and selection of the best development strategy will support Harbin City’s sustainable growth. (2) The proportion of people in Harbin living in urban areas is 70.61%, markedly exceeding the country’s average proportion of 63.89%. This rapid urbanization is accompanied by resource depletion and environmental pollution. In Harbin, the quality of the environment for wild animals and plants has declined, the amount of organic matter in the soil has dropped, and the city is subjected to several tests for solid, water, and air pollution. There have been considerable disasters (droughts, floods, and other calamities) over the past few years. It is necessary to investigate the logical planning direction for the future green space pattern to optimize the ecological environment and boost the development of an ecological civilization in Harbin. The future green space pattern in Harbin urgently needs to be replanned, and it must serve as an example for other cities with a similar scenario due to the tension between the city’s high standing in politics, economics, and culture and the severe state of the natural environment.

### 2.2. Data Source

The land cover data for 2010 and 2020 were sourced from National Catalogue Service for Geographic Information (http://www.globallandcover.com/ accessed on 1 January 2023); they were blacked out, merged, and cropped in ArcGIS. The study region’s land cover types included cultivated land, forest, grassland, wetland, water area, artificial surface, and bare land, all of which were classified by the Globelland30 system. 

Table 1 lists the multiple driving factor data employed in this study, including natural drivers, social drivers, and traffic drivers. DEM data originated from Geospatial Data Cloud (www.gscloud.cn accessed on 1 January 2023); they were merged in ArcGIS and then extracted by mask. While other natural parameters (e.g., the average annual temperature and precipitation data) were collected using the inverse distance weighting method, slope and aspect data were derived using DEM data in ArcGIS. Social drivers comprised population density and per capita GDP kilometer grid data, which were sourced from World Pop (https://www.worldpop.org/ accessed on 1 January 2023) and Geographical Information Monitoring Cloud Platform (http://www.dsac.cn/ accessed on 1 January 2023), respectively. Traffic drivers originated from National Catalogue Service for Geographic Information (https://www.webmap.cn/ accessed on 1 January 2023), including distances from the river, national highways, highways, high-speed roads, county roads, and railways, and the distributions of distance variables were determined through Euclidean distance. The data from Harbin City Nature Reserve were selected and then rasterized in the Resource and Environment Science and Data Center (https://www.resdc.cn/ accessed on 1 January 2023), and they served as a limiting factor. In accordance with the experiment, the resolution was determined as 100 m × 100 m by consulting a considerable amount of relevant research [23,33,68] to ensure that the model can operate effectively. To ensure data consistency, WGS_1984_UTM was selected as a unified coordinate system, in which the same number of rows and columns were set for the driver factor data, and normalized processing was carried out.

### 2.3. Research Procedures

**Figure 2:**
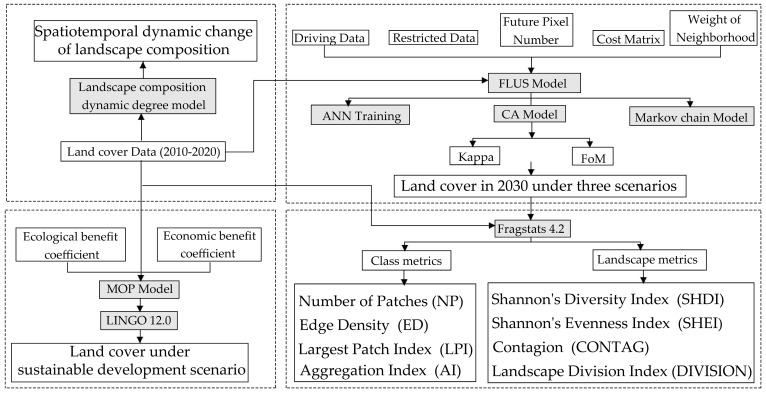
Technical flow chart.

This study was conducted in four sections, as illustrated by the flow chart in Figure 2. In the first step, the landscape composition dynamic degree model was employed to determine the dynamic degree indices and examine the dynamic spatiotemporal changes in the research region between 2010 and 2020. In the second step, the distribution of land cover types in several scenarios in 2030 was modeled and predicted using the GeoSOS-FLUS software in accordance with characteristics (e.g., land use demand, transfer cost matrix, and neighborhood factors). In the third step, the landscape pattern was compared using Fragstats4.2 in three different scenarios, the temporal and geographical variations in the landscape pattern were evaluated, and the landscape pattern index at the type level and the landscape level were determined using the landscape pattern index method. Lastly, the MOP model was adopted to determine the optimal quantitative structure of the LULC types with the goal of balancing ecological benefits and economic benefits. 

### 2.4. Landscape Composition Dynamic Degree Model

Landscape composition dynamic degrees (i.e., single landscape composition dynamic degrees and comprehensive landscape composition dynamic degrees) were adopted to express the rate of regional land use change, deepen the comparison of regional differences, and forecast dynamic change trends of future LULC. Single landscape composition dynamic degrees represent the direction and rate of change in a certain LULC type in a unit period; the formula is expressed as follows:

(1) $$K=\frac{U_{b}-U_{a}}{U_{a}}\times \frac{1}{T}\times 100\%$$

where *K* denotes the dynamic degree of a single landscape composition; *U_a_* represents the area of a certain land cover type at the beginning of the study period; *U_b_* expresses the area of a certain land cover type at the end of the study period; and *T* is the time range of the study, in units of years.

The transfer of all land cover types across the entire study region is indicated by the comprehensive landscape composition dynamic degrees. The more severe the research area’s changes in land cover types, the higher the value will be. The formula is written as follows:

(2) $$L_{c}=\frac{\sum_{i=1}^{n}\Delta LU_{ij}}{2\times \overset{n}{\underset{i=1}{\sum }}LU_{i}}\times \frac{1}{T}\times 100\%$$

where *L_c_* denotes the comprehensive dynamic degree of land cover type C; *LU_i_* is the area of land cover type i in the initial period; Δ*LU_ij_* is the area of land cover type i transformed into type j; and *T* is the time range of the study, in units of years.

### 2.5. Land Cover Change Simulation Model

#### 2.5.1. Model Selection and Introduction

The study area was simulated using the GeoSOS-FLUS software based on the land cover data in 2010 and 2020, and the land cover change in the three scenarios in 2030 was predicted. The FLUS model of GeoSOS-FLUS software comprised three modules (i.e., top-down system dynamics (SD) model, bottom-up cellular automata (CA) model, and an artificial neural network (ANN) model). Moreover, a roulette selection mechanism was introduced [69]. Furthermore, a Markov chain model was covered in the software to facilitate the estimation of future land use requirements.

#### 2.5.2. FLUS Model Parameter Setting 

##### Calculation of Suitability Probabilities

The possibility that each land cover type will be present in each cell is known as the suitability probability. The research area’s land cover data from 2010 were imported into the model as the fundamental data, and the random sampling strategy was chosen. The number of sampling parameters was set to 20, and the number of hidden layers in the neural network was set to 12. Then, the normalized driver data were then imported into the model using the same row and column numbers. The suitability atlas was eventually obtained under the limiting constraint, and the root mean square error was 0.21. In this study, the suitability probabilities were calculated using the following formula:

(3) $$\sum_{k}p\left(p,k,t\right)=1$$

(4) $$p\left(p,k,t\right)=\sum_{j}\omega_{j,k}\times sigmoid\left[net_{j}\left(p,t\right)\right],\;=\sum_{j}\omega_{j,k}\times \frac{1}{1+e^{-net_{j}(p,t)}}$$

where *p (p*, *k*, *t)* denotes the suitability probability of the land cover type k in the period *t* and the grid is *p*; $\omega_{j,k}$ is the weight of the output layer and the hidden layer; sigmoid () represents the function of the hidden layer and the output layer; *net_j_ (p*, *t)* is the signal received by the *j*th hidden layer grid *p* at time *t*.

##### Neighborhood Factor Parameter

The neighborhood factor parameter, which is proportionate to how well LULC kinds may expand on their own, represents the interaction between different land types. The parameter’s range is 0 to 1, and the closer it is to 1, the easier it is to transform and the more capacity for expansion the related land type has. Since it is challenging to quantify LULC types’ expansion capacity directly, the LULC types were first processed in a dimensionless manner. Subsequently, they were tested numerous times under a wide variety of scenarios, and the experimental findings were compared to yield the final results. The specific formula for dimensionless processing is as follows:

(5) $$X*=\frac{X-\min}{\max-\min}$$

where X* denotes the normalized value of the deviation; min represents the minimum value of the data; max expresses the maximum value of the data.

##### Conversion Cost Matrix

The criteria for reciprocal conversion between LULC kinds are referred to as the conversion cost matrix. When the cost matrix has a value of 1, conversion is possible; when it has a value of 0, conversion is not possible. The conversion cost matrix for this study was created considering the scenario’s requirements as well as the features of changing land cover in the study area between 2010 and 2020.

##### Accuracy Verification

The simulated land cover data for 2020 were compared with the actual land cover data for 2020 based on the real land cover data from 2010, and when combined with the driving factors, the Kappa coefficients were validated and the overall accuracy (OA) was computed [70]. The model is considered to have a high level of confidence as well as being usable when the Kappa index is more than 0.75. The accuracy of the simulation is stated as being higher the closer the overall accuracy (OA) is to 1. The FLUS model has a good impact on the land cover simulation of Harbin in 2030 and has excellent practicality, as shown by the research’s OA coefficient of 91.55% and Kappa coefficient of 0.86.

### 2.6. Scenario Descriptions

Four simulation scenarios were set in accordance with the trends of the study area’s future development scenarios and relevant policies.

- The status quo development scenario, as with the 2010–2020 LULC transfer scenario, does not take into account the effects of pertinent national policies and does not impose any conversion restrictions. The neighborhood factor parameters and transfer cost matrix were kept the same in this scenario, which was primarily a continuation of the land use change trend from 2010 to 2020.
- In the ecological protection scenario, priority was given to the preservation and improvement of the ecological environment. The 14th Five-Year Plan of Harbin states that improvements should be made in the ecological environment’s quality, the green development system, the protection of forest resources, and the preservation and restoration of rivers, lakes, and wetlands. This scenario was developed on the LULC transfer matrix of the status quo development scenario, where urbanization development was restrained and natural ecosystems (e.g., woods, wetlands, and water areas) were significantly safeguarded. The constraint condition is to give priority to the benefit of the ecological environment while reducing the transfer probability of forest land to the artificial surface to 50%, reducing the transfer probability of cultivated land to the artificial surface to 30%, and increasing the transfer probability of cultivated land to woodland and grassland to 20%.
- In the economic development scenario, the development of the city’s economy should be prioritized. The study region has a significant economic significance as Northeast China’s northern economic hub and the beginning of the Ha-Da-Qi Industrial Corridor. The requirement to energetically grow the real economy, establish a regional innovation highland, and concentrate on high-quality development was stated in the 14th Five-Year Plan and the Harbin City 2035 Plan. The natural environment would also be somewhat impacted at the same time. The artificial surface would be increased in accordance with the evolution of status quo scenarios, and other areas such as forests and grasslands would be decreased. The constraint condition is to give priority to the benefit of economic development, increasing the conversion probability of woodland and grassland to artificial surface by 50% and the conversion probability of woodland to cultivated land by 30%.
- The economic position and the growth of the ecological environment were considered in the sustainable development scenario. Maintaining the ecological environment cannot be disregarded during the growth of the economy, and the slowing down of economic progress by excessive ecological protection should be avoided. The economic benefit target and the ecological benefit target should reach the maximum values simultaneously, such that the final overall benefit is the highest.

### 2.7. Landscape Pattern Index Method

The landscape pattern index may quantify the spatial properties of a landscape pattern, represent its structural makeup, and show its temporal and spatial changing trend [71,72]. The traits of land cover landscape patterns are primarily evaluated from three separate perspectives (i.e., patches, classes, and landscapes) [73]. In this study, the landscape pattern of Harbin City was investigated at the class and landscape levels using the Fragstats4.2 software. At the class level, the number of patches (NP), edge density (ED), largest patch index (LPI), and aggregation index (AI) were selected; at the landscape level, Shannon diversity index (SHDI), Shannon evenness index (SHEI), contagion index (CONTAG), and landscape division index (DIVISION) were selected. Table 2 lists the landscape pattern indices and their ecological meaning.

### 2.8. MOP Model

The multi-objective programming (MOP) model comprised three parts, i.e., decision variables, constraints, and objective functions. The fundamental idea behind the model is to specify the objective function and constraint conditions so that the optimal value for the decision variables may be determined. It maximizes the advantages to be attained while optimizing the structure of land cover quantity. 

#### 2.8.1. Set Decision Variables 

Seven different types of land cover areas served as the decision variables to construct the model in accordance with the Globelland30 classification system and the green space classification system mentioned in this study: x_1_ = cultivated land, x_2_ = forest, x_3_ = grassland, x_4_ = wetland, x_5_ = water area, x_6_ = artificial surface, x_7_ = bare land.

#### 2.8.2. Set up Objective Functions

Two optimization objectives—ecological benefit and economic benefit—were chosen based on the features of the study area and the difficulties of data quantification. The ecological benefit objective function (6) and economic benefit objective function (7) are as follows:

(6) $$E_{p\left(x\right)}=\sum_{i=1}^{n}p_{i}\cdot x_{i},$$

(7) $$E_{d\left(x\right)}=\sum_{i=1}^{n}d_{i}\cdot x_{i},$$

where *E_p(x)_* and *E_d(x)_* denote the ecological benefit and economic benefit, respectively; *x_i_* represents the land type *i* variable (*i* = 1, 2, 3, 4, 5, 6, 7); *p_i_* and *d_i_* express the ecological and economic benefit coefficients per unit area of the land cover type *i*. 

The equivalent factor method was adopted to determine the ecological benefit coefficient (CNY 1 million/km^2^) of each land cover type based on the Statistical Yearbook, the “Compilation of National Agricultural Product Cost and Benefits Data”, and the equivalent table of ecosystem service value per unit area examined by Xie et al. [74,75,76]. The ecological benefit of the artificial surface is almost zero this is where most people live and conduct their daily activities. Thus, its ecological benefit coefficient was set at 0. The average land economic value (CNY 1 million/km^2^) of each type of land cover in 2020 can be determined by checking the statistical yearbook of the study area. This value can be used to calculate the economic benefit coefficient of each type of land. The economic benefit coefficient of bare land was set to zero because of the minimal amount of bare land and its extremely low economic benefits. The final economic benefit objective function (8) and ecological benefit objective function Formula (9) are as follows:

(8) $$E_{p\left(x\right)}=86.78x_{1}+432.73x_{2}+271.97x_{3}+1142.82x_{4}+1492.56x_{5}+0x_{6}+4.39x_{7}$$

(9) $$E_{d\left(x\right)}=284.18x_{1}+17.09x_{2}+749.28x_{3}+123.90x_{4}+125.98x_{5}+20261.03x_{6}+0x_{7}$$

The optimal distribution of land cover quantity structure requires the maximization of these two objectives at the same time; the formula is as follows:

(10) $$\max\left\{E_{d\left(x\right)},E_{p\left(x\right)}\right\}=\alpha E_{d\left(x\right)}+\beta E_{p\left(x\right)}\max\left\{E_{d\left(x\right)},E_{p\left(x\right)}\right\}=\alpha E_{d\left(x\right)}+\beta E_{p\left(x\right)},$$

where $\max\left\{E_{p\left(x\right)},E_{d\left(x\right)}\right\}$ denotes the comprehensive maximum value of ecological benefits and economic benefits. According to the future development orientation of the study area and expert opinions, the parameters *α* = 0.60 and *β* = 0.40 were set. Lastly, LINGO12.0 was used to solve the above formulas combined with constraints.

#### 2.8.3. Build Constraint Condition

Constraints are established with reference to the level of the land cover area in the research area in 2020, as indicated in Table 3 below, to ensure that future land cover changes comply with the law of natural development.

## 3. Results

### 3.1. Dynamic Degree of Green Space Landscape

**Figure 3:**
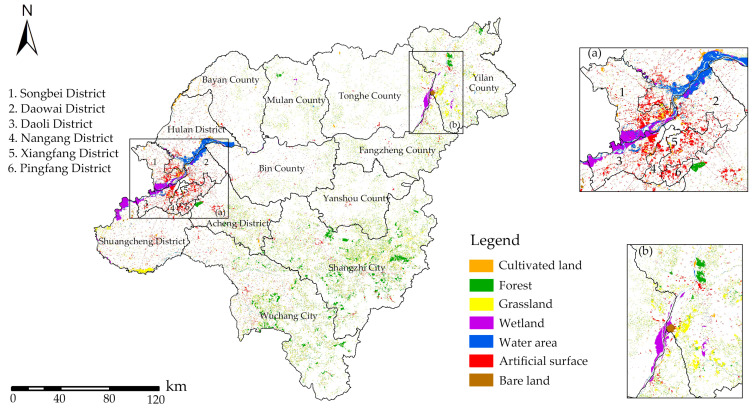
Land cover gain from 2020 to 2010. (**a**) Land cover gain in urban Harbin from 2020 to 2010. (**b**) Land cover gain at the junction of Tonghe, Yilan and Fangzheng counties from 2020 to 2010.

**Figure 4:**
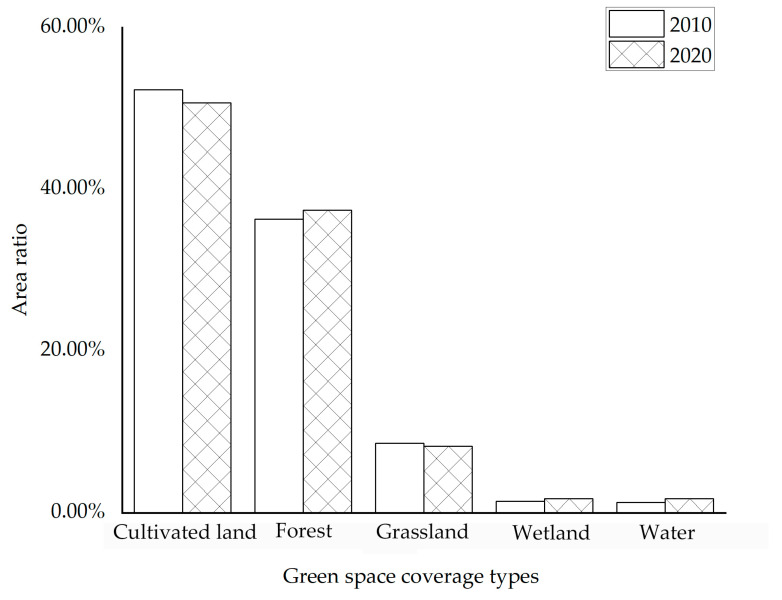
The proportion of green space coverage in 2010 and 2020.

**Figure 5:**
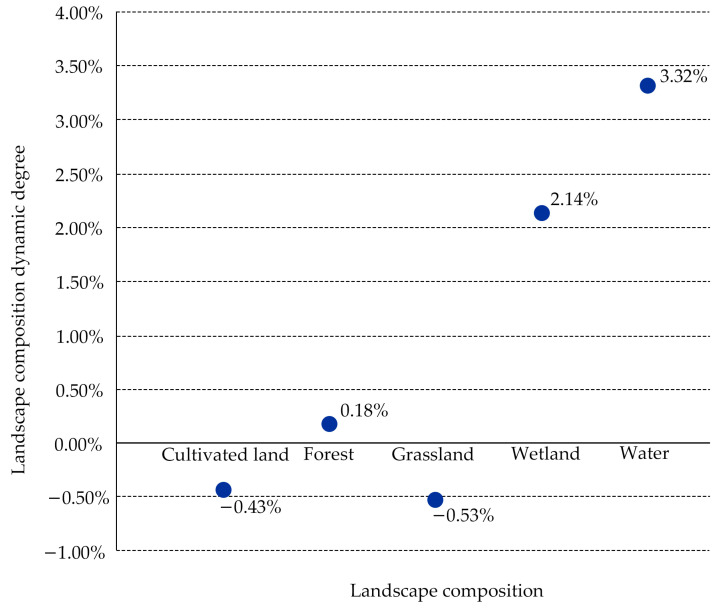
The landscape composition dynamic degree in the study region from 2010 to 2020.

In the period between 2010 and 2020 (Figure 3), wetlands and waters in the Harbin metropolitan area increased significantly, and grasslands and woodlands increased slightly within the city limits. According to the research, just a minor portion of the total green space area was made up of wetlands and water (Figure 4). In addition, cultivated land, which made up more than 50% of the total area, was the predominant form of green space landscape, followed by forest and grassland, which made up more than 35% and 8%, respectively. The landscape dynamics of cultivated land, forest, and grassland changed relatively slowly during the past 10 years—all by less than 1%, as indicated in Figure 5. The cultivated land area and the grassland area shrunk by 1169.51 km^2^ and 239.20 km^2^, respectively. Wetland and water areas changed relatively quickly, with wetlands changing by 2.14%, while the water area’s degree of dynamic change (3.32%) was the greatest of all the different types of green space landscapes, and both developments were positive. This was mostly due to the research area’s significance placed on the preservation and development of wetlands and water resources, both of which were interdependent and supportive of one another. Only 0.20% of the research area’s comprehensive landscape composition was dynamic between 2010 and 2020, and change there was also significantly slow.

### 3.2. Future Green Space Pattern in Multiple Scenarios

**Figure 6:**
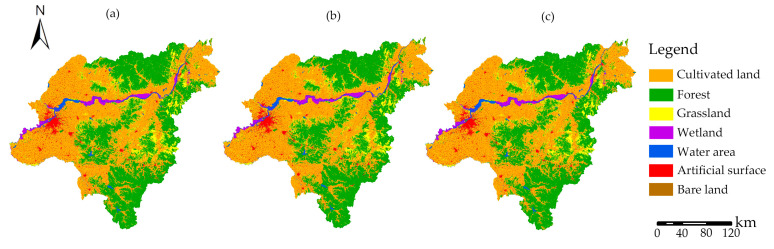
Land cover under different scenarios in Harbin in 2030. (**a**) Land cover in status quo development scenario; (**b**) land cover in ecological protection scenario; (**c**) land cover in economic development scenario.

**Figure 7:**
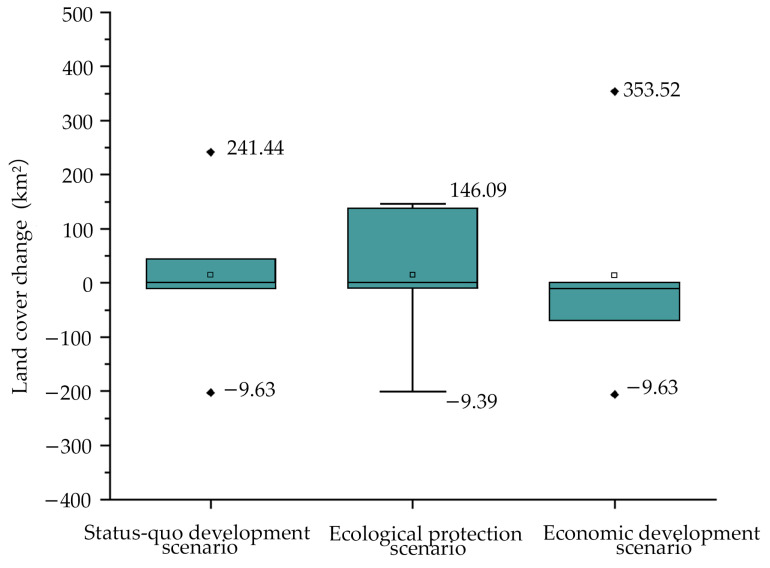
Land cover change under different scenarios in Harbin in 2030.

The distribution pattern of Harbin City under the three FLUS model-simulated scenarios is shown in Figure 6. The major land cover type in the three scenarios is cultivated land, which also experiences a slow increase in wetland areas and a decline in water areas (Figure 7). Compared with the green spatial pattern in 2020, the cultivated land and forest land have obvious changes.

In the status quo scenario, it is anticipated that cultivated land and forest land will increase by 241.44 km^2^ and 44.01 km^2^, respectively, while grassland is expected to shrink by 202.90 km^2^. This is mainly indicated in the triangle area surrounded by Yuquan Tiger Mountain Forest Park, Xiquanyan Reservoir Tourism Area, and Jinlong Mountain International Tourism Resort; the northwest corner of Shangzhi City; and the area between Changshou Mountain and Shuangyazi Mountain in Yanshou County. The water area and wetland will remain relatively stable. The city area’s ecological environment is effectively safeguarded in the ecological protection scenario, and the growth in the forest is the highest among the three scenarios, with an estimated increase of 137.46 km^2^, which is expected to be 3 times the status quo increase. The total increase in cultivated land is anticipated to be 146.09 km^2^, and this is the smallest increase of the three, less than 50% of that of the economic development scenario. This increase is mainly manifested in the southeast of Acheng District and the northwest of Shangzhi City. It can be seen that the policy and measures of returning farmland to the forest have been effectively maintained in this scenario. In the scenario of economic development, cultivated land is in a trend of rapid expansion and is anticipated to rise by 353.52 km^2^ to ensure quick economic growth. The artificial surface’s downward tendency has lessened. With an expected reduction of 70.97 km^2^, the economic development scenario has the least area of artificial surface reduction in comparison with the other two scenarios. Consequently, there is a significant impact on the ecological environment. The area of grassland is anticipated to shrink by the greatest amount, to 206.17 km^2^, and the area of forest is anticipated to decrease by 69.19 km^2^ in this scenario. The intersection between Acheng District and Shangzhi City, the southeast corner of Wuchang City, and the Songhua River basin of Yilan County were the key locations where forest land underwent alterations.

### 3.3. Characterization of Changes in Green Space Landscape Pattern Index

#### 3.3.1. Characterization of Changes at the Class Level

**Figure 8:**
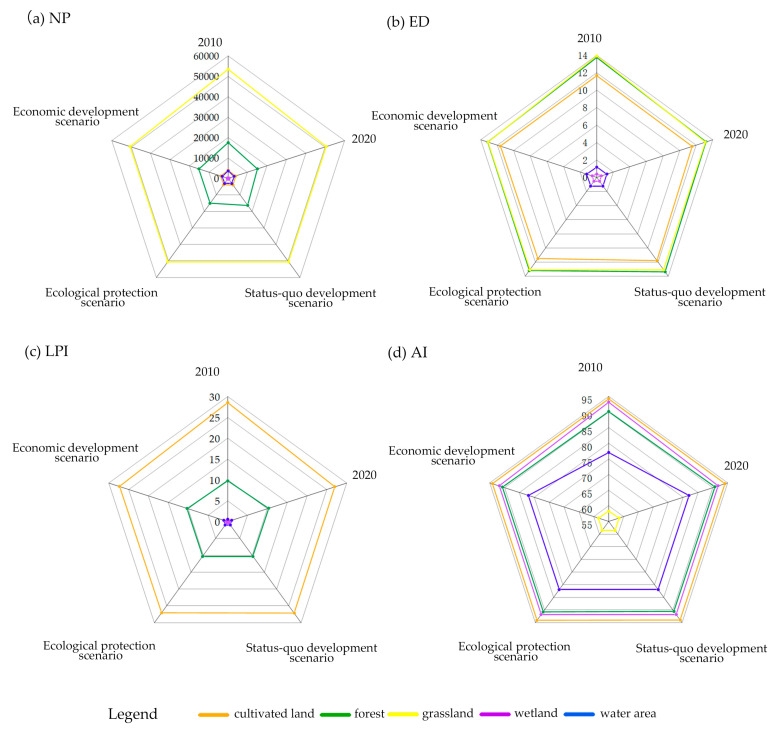
Class-level landscape indices in the study region.

According to the class-level study (Figure 8), NP and ED indicate the degree of landscape fragmentation in the study area, showing landscape heterogeneity and patch edge segmentation, which are positively correlated with the degree of fragmentation [77]. From 2010 to 2020, NP and ED in wetland areas showed an increasing trend, while those in cultivated land, forest land, and grassland showed a decreasing trend. Cultivable land had the smallest decline, with NP falling by 171, followed by water area, falling by 574. During the period, NP declined by 2882 in grassland, which represented the biggest decline. Grassland ED likewise experienced the highest decline, falling by 0.80, despite the fact that ED’s overall variation trend was modest. This indicates that wetland protection schemes were evidenced to not have been fully implemented or enforced, with less fragmentation of cropland, woodland, and grassland, as well as a steady simplification of edge patterns. In the three scenarios in 2030, the NP values of cultivable land and grassland increased in comparison with 2020, mainly due to human development activities, and the NP and ED of wetland areas remained stable. Wetland had the least amount of fragmentation compared to other types of green space coverage, with an estimated NP of 500 and an expected ED of less than 0.60. In the ecological protection scenario, the patch numbers of cultivated land and grassland were the smallest among the three scenarios, and the increase in patches in both scenarios was not expected to exceed 20. The edge density of the water area to remain steady. The cultivated land edge density rise trend is predicted to be the least, and the changing trend is predicted to be less than 0.63% of that of the status quo development scenario. The main reason was that in this scenario, the fragmentation of cultivated land was effectively controlled, and relevant policies of ecological protection were well implemented. In the economic scenario, the densities of forest land, grassland, and water edge were lower than those in 2020. The NP of grassland in this scenario, even though it only rose by 49, was 2.72 times greater than that in the ecological protection scenario and twice as much as that in the status quo scenario, showing that this scenario’s patch boundaries tended to be regularized and the grassland fragmentation was the worst.

LPI is a measure of how much of the landscape’s overall area corresponds to the size of the largest patch. It is conducive to determining the dominant type of landscape and indicates the direction and strength of human activities. The LPI of cultivated land and forest was notably higher than that of other green space coverage landscape types, suggesting that cultivated land and forest were the most prevalent and dominant land cover types in the studied area. The patch index of cultivated land continued to be the largest from 2010 to 2020, despite a decreasing trend in the LPI of cultivated land and an increase in most of the remaining green space coverage landscape types. The rate of rise for grassland and wetland was less than 0.02%, while the rate of increase for forest land was the highest but was limited to 0.60%. In the three scenarios, it is anticipated that the LPI for grassland, wetlands, and water will remain essentially unchanged in 2030 from its value in 2020. The maximum patch index of cultivated land is expected to rise in the economic scenario, whereas it is already nearly 70 times that in the ecological protection scenario. In addition, the maximum patch index of forest land decreased significantly, which was 4 times that of the status quo development scenario. The primary reason for the above-mentioned result is that agriculture is intensively developed in this scenario for economic development. For the development of the economy and the expansion of agricultural development, the forest ecosystem has been destroyed, and the principle of returning farmland to the forest has been violated.

AI represents the randomness of different patch types. The more dispersed the patches are, the smaller the aggregation index will be. In addition, the aggregation index is increased with patch size and reaches its maximum of 100. The forest, grassland, wetland, and water area aggregation indices all displayed an upward trend from 2010 to 2020, showing that the patches of these green space-covered landscape types are growing more and more aggregated. The greatest increase, from 77.04% to 81.94%, was seen in the water area. The cultivated land is becoming more and more dispersed, whereas it has the greatest AI in comparison with other types; i.e., all of it is above 94.07%. The three scenarios are projected to result in a declining trend in the amount of cultivated land, forest, and grassland relative to 2020, with the ecological protection scenario exhibiting the least amount of reduction, and with a projected decline of less than 0.01% for cultivated land and forest land, indicating less division and interference with these resources. Wetlands and water areas are expected to increase slightly, and the relative growth may be rather high in the economic development scenario. This is primarily because of economic needs (e.g., the growth of water area tourism). Table 4 lists the landscape pattern indices at the class level.

#### 3.3.2. Characterization of Changes at the Landscape Level

**Figure 9:**
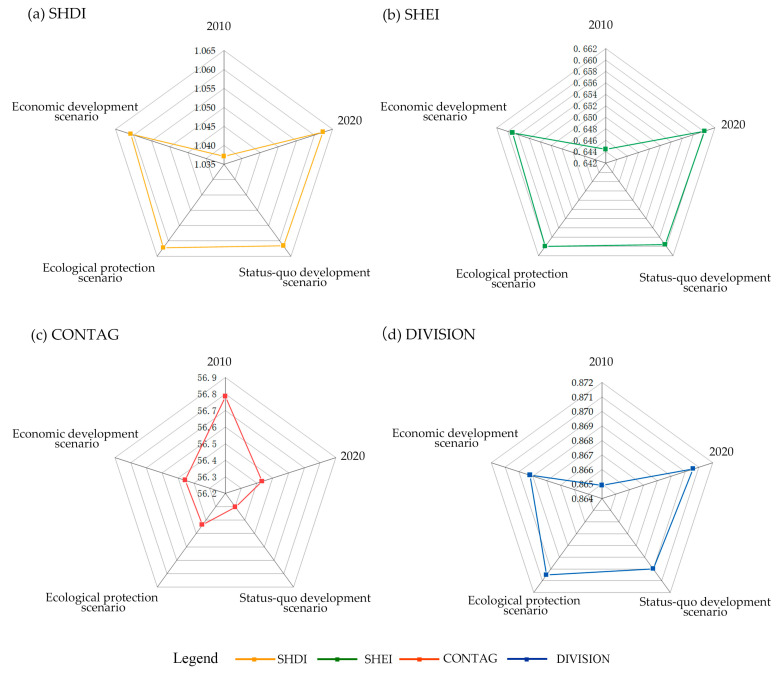
Landscape-level landscape indices in the study region.

Analysis at the level of the entire landscape is shown in Figure 9. The richness and uniformity of the landscape are expressed by SHDI and SHEI, respectively. The type of scenery exhibited improved richness and uniformity with an increase in these values. Both indices rose between 2010 and 2020, suggesting that the level of the general landscape progressed toward diversification and homogenization during this time and that the degree of interconnectedness between them rose. The SHDI and SHEI are expected to decrease by the greatest amount in the economic development scenario, 8 times more than in the ecological conservation scenario, and both decreases are smaller than 0.01. As indicated by the above-described results, economic development was overemphasized and artificial land was developed such that the landscape’s fragmentation was worsened and the uniformity of distribution was affected. The dominating patch’s extension trend and degree of agglomeration are referred to as CONTAG; the greater the CONTAG value, the more unbroken the landscape will be, with good connectivity. The CONTAG value fell between 2010 and 2020, suggesting that there was less connectedness between cultivated land and forest. In the status quo scenario, the CONTAG was expected to keep declining, whereas the range was narrow, less than 0.13%. In both the ecological and economic scenarios, the CONTAG is predicted to marginally rise—less than 0.03% in each case. Particularly, it is anticipated that the CONTAG will increase more in the economic development scenario and will be 7 times more than it would be in the ecological protection scenario. It was largely attributed to the value placed on the development of agricultural cultivated land. DIVISION indicates the degree of separation of patches in the landscape; the larger the value, the more fragmented the patch composition and complicated the landscape will be. The DIVISION index in 2020 increased by 0.01% compared with that in 2010, indicating that patches tend to be fragmented in the landscape during this period. Compared with 2020, the DIVISION in 2030 is expected to slightly decline. Among the three scenarios, the DIVISION may have the largest decline in the economic development scenario. The major reason for this result is that cultivated land is the dominant land cover type with the largest area, thus facilitating economic improvement. During the above-described period, cultivated land will be primarily developed to maintain economic benefits. Table 5 lists the landscape pattern indices at the landscape level.

### 3.4. Green Space under the Sustainable Development Scenario

The areas of x_1_~x_7_ under the sustainable development scenario are calculated as 22,299.88, 19,379.10, 4271.00, 920.74, 4648.54, 2453.68, and 17.24 km^2^ using the MOP calculation model and LINGO software. In comparison to 2020, it is anticipated that the area of cultivated land in green space would decrease by 3935.27 km^2^, while the area of water will increase by 223.06 km^2^, and the area of other types of green space will remain stable. Table 6 demonstrates that while the bare land declines by 0.07 km^2^, the artificial surface in non-green space expands by 3712.28 km^2^. The ecological benefit of the ecological protection scenario was the highest at CNY 142,776.51 million, the economic benefit of the economic development scenario was the highest at CNY 219,676.57 million, and the comprehensive benefit of the ecological protection scenario was the highest at CNY 361,891.06 million for all three scenarios in terms of status quo development, ecological protection, and economic development. The ecological benefit is anticipated to total CNY 194,731.82 million, the economic benefit is anticipated to total CNY 241,129.06 million, and the overall benefits are expected to total CNY 435,860.88 million in the sustainable development scenario. The sustainable development scenario exceeds all other development scenarios in terms of both ecological and economic benefits, as shown in Table 7.

## 4. Discussion

### 4.1. Green Space Landscape Pattern Index

In terms of time dynamics, from 2010 to 2020, the fragmentation degrees of cultivated land, forest land, and grassland decreased, and the edge shape was gradually simplified. Cultivated land, the most dominant land cover type, became increasingly dispersed in this period, whereas the concentration of cultivated land was the highest among all green space types. In general, the green spatial pattern tended to be diversified and uniform, and it was more significantly connected. However, the two dominant land cover types, cultivated land and forest, had decreasing connectedness and somewhat increased patch fragmentation in the landscape, respectively. From 2020 to 2030, most of the green spatial patterns will be more fragmented, and the landscape heterogeneity will decline. The change in wetland areas is excepted be slight, such that the status of 2020 was nearly unchanged. The above-mentioned findings are consistent with those of He et al. [78] who investigated changes in the Songhua River Basin in Harbin’s landscape patterns. 

In accordance with several scenarios, the status quo development scenario is projected to result in less patch aggregation of cultivated land, grassland, and forest, regularization of the margins of grassland and water regions, and a reduction in the fragmentation of water areas. The ecological protection scenario stresses the protection of the ecological environment, and the fragmentation of cultivated land, grassland, and forest land is expected to be slightly increased, significantly lower than the number of patches in the status quo scenario. In this scenario, it is anticipated that the maximum patch index will rise and that the diversity and homogenization of the landscape will both decline less than in the other two scenarios, consistent with the conclusions of previous research [25,79]. In the economic development scenario, the connectivity of cultivated land is expected to increase for the development of the agricultural economy. Wetland and water aggregation are expected to be slightly improved for the development of water tourism. Serious fragmentation of forest patches is expected to result from economic development, similar to the findings of Zhang et al. [80].

### 4.2. Green Space Optimal Scenario

In this study, the land cover model in 2030 was simulated and forecasted using the FLUS model in combination with the Markov chain model in accordance with the land cover type data in 2010 and 2020. Moreover, the optimal sustainable development scenario was calculated by combining the MOP model and the LINGO function.

The forest land in the green space was continuously protected in the status quo development scenario, and it will tend to expand and gain numerous ecological benefits. In the ecological protection scenario, the preservation of the ecological environment is prioritized, and economic development is constrained to prevent further harm and exploitation of the environment. Accordingly, in this scenario, it is anticipated that the forest will grow rapidly, the cultivated land will grow less, and the loss of grassland and water regions will be inhibited, consistent with the findings of earlier studies [81]. Economic benefits are put first in the economic development scenario, and artificial surfaces should be developed and constructed for market trading. The shift in the amount of green space covered in this scenario clearly reveals that agriculture expansion is effective in boosting the local economy. Shi et al.’s findings [82] are consistent with the findings of this study since the living environments of both grassland and forest land were destroyed simultaneously, and the ecological advantages were significantly impaired when substantial economic benefits were being received. The ecological preservation scenario achieved the largest overall benefit of the three, whereas its economic gain was relatively quite low, thus hindering the standard improvement of living for people. As a result, the MOP model was employed to determine the optimum course of action for the study. The study’s findings suggest that the decreasing amount of farmed land, the increasing amount of water and artificial surfaces, and the maintained condition of other green spaces are considered the optimal conditions for the sustainable growth of the study area. It is noteworthy that in this scenario, ecological and economic advantages will be maximized. In brief, the cultivated land and the forest take on a major significance for green space, with the development of cultivated land being biased towards the economic level and the effect of forest land being biased towards the level of environmental protection in accordance with changes in the coverage of green space and their benefits in the scenarios of natural development, ecological protection, and economic development. As revealed by the scenario for sustainable growth, the water area is also crucial and requires active maintenance. The key to directing the sustainable development of the research area is that the country should facilitate the implementation of the policy of returning cultivated land to forests, implement the maintenance of forests, place stress on the development of water resources, and tap the inherent potential of cities while limiting disorderly urban expansion. The scenario of balanced ecology–life–production space development suggested by Zhao et al. [39], with simultaneously maximized ecological and economic benefits, is consistent with the findings of this study. 

### 4.3. Research Significance at Home and Abroad

This study compared green space landscape patterns of different scenarios in the study area in the future. The related benefits were analyzed, which can be conducive to providing direction for policy formulation in the study area and development reference for similar cities. This analysis makes up for the deficiencies in the study of Northeast China. Moreover, the in-depth analysis of this study reveals that the connectivity and fragmentation of green spatial patterns at the landscape level are largely determined by the dominant land cover type. In the research area, cultivated land is the dominant land cover type. The degree of overall landscape fragmentation of the cultivated land is smaller in the economic development scenario than it is in the ecological preservation scenario, and the ecological benefits at this time are extremely minimal. Accordingly, this study can provide theoretical support for the study of urban landscape patterns with cultivated land as the dominant land cover type. To be specific, this study compares multiple scenarios using a combination of qualitative and quantitative approaches, as well as a variety of technical techniques and municipal planning laws. The above-mentioned method is capable of providing ideas for sustainable development planning at home and abroad.

### 4.4. Uncertainties and Implications

However, there are some uncertainties in this study. The first limitation of this study is the precision of the land cover data. The 2020 data were primarily 16 m resolution Gaofen-1 multispectral photos, whereas the GlobeLand30 data were primarily 30 m multispectral images. There were variations in their precision due to technology’s constant advancement. The overall accuracy of the 2010 data reached 83.50%, with a Kappa coefficient of 0.78, whereas the overall accuracy of the 2020 data reached 85.72%, with a Kappa coefficient of 0.82. The two different levels of precision could cause a tiny disparity between the study’s findings and the reality. The conclusions drawn from existing research will cause biases since there is currently no comprehensive and universal framework for evaluating the value of ecosystem services. Therefore, the establishment of ecological and economic benefit indicators and the improvement of ecosystem service value evaluation methods are hotspots for future research.

Furthermore, this study has certain shortcomings; we hope that these can be fixed in subsequent research. In this study, the FLUS model was used to simulate the green space coverage types in Harbin in three scenarios. It should be noted that the corresponding driving factors and some scenario setting parameters need to be set in the simulation of the FLUS model, and this study’s driver settings were not sufficiently complete. The selection of driving variables lacked the effect of policy elements (e.g., ecological protection red lines and urban development limits) on the future land cover due to the inadequacies in the drafting of pertinent policies. In addition, the selection and parameter setting of other driving factors are subjective, such that the coverage distribution of green space is inaccurate [29,83]. Therefore, how to get rid of subjectivity, choose driving factors and parameter settings more objectively, follow up with policy support, and integrate findings into policy scenarios to obtain more accurate future land cover situations should be further explored in depth.

In this study, the status quo development scenario, the ecological protection scenario, and the economic development scenario were simulated using the FLUS model, whereas these are not all possible scenarios in the future. In addition, it is noteworthy that development priorities and consideration factors vary with periods. To make more thorough policy recommendations, future studies should begin with a variety of viewpoints and development scenarios, including an analysis from the following perspectives: First, low-carbon development scenarios can be set. Climate change is an important global environmental issue [84]. Faced with the global climate issue, China has proposed a significant strategy to achieve a carbon peak by 2030 and carbon neutrality by 2060 [85,86]. Therefore, controlling carbon emissions and creating a low-carbon environment is the future direction of development. In addition, the cultivated land protection scenario can be considered. The security of the nation’s cultivated lands serves as both the cornerstone and the lifeblood of its sustainable growth [87]. To guarantee the area of cultivated land, this scenario strictly implements the additional policy of cultivated land [88].

According to this study, the main difference in green space landscape patterns in the three scenarios is reflected in cultivated land and forest land. The sustainability scenario study’s findings indicate that limiting the growth of arable land and converting it into water and non-green land as much as possible, which reduces the invasion of forest land, is the primary option to achieve the highest combined benefits. However, this green space pattern may have an impact on people’s dietary needs. We might also start with the following factors to create sustainable growth in Harbin: First of all, advanced farming techniques can be introduced and scientific planting methods can be used to increase the agricultural yield per unit area [89,90]. Secondly, explore the new development mode of forests, wetlands, grasslands, etc.; shape the endogenous development power; enhance their economic value through innovative technological means; and relieve the economic pressure of cultivated land and water area [91]. Finally, high and new technologies can be introduced to promote the upgrading of industrial structure, optimize the energy structure, and increase the economic efficiency of non-green space to reduce the economic demand for cultivated land. [92].

## 5. Conclusions

The dynamic attitude of green space composition in Harbin from 2010 to 2020 was calculated in this study. Subsequently, the green space in 2030 was modeled using GeoSOS-FLUS software in accordance with the views of status quo development scenarios, ecological preservation scenarios, and economic development scenarios. Next, the changes in landscape pattern index in 2010, 2020, and three different scenarios were compared and analyzed. Lastly, based on multi-objective conditions, the land cover needs in the ideal sustainable development scenario were determined using the MOP model. This study has filled the gap in the research of the cities in Northeast China and provides a reference for the future development of Northeast China and similar cities. The results are summarized as follows:

From 2010 to 2020, the dynamics of the landscape’s cultivated land, forests, and grasslands experienced a slow change, whereas wetlands and water areas showed quick and beneficial changes. Moreover, the total landscape composition’s dynamic degree, which merely reaches 0.20%, has changed quite slowly.

The primary landscape type in the three 2030 scenarios is cultivated land. The status quo scenario anticipates a rise in cultivated land and forest, a decline in grassland, and little to no change in water bodies and wetlands. The increase in forest land is expected to be the largest in the ecological protection scenario, while the rise in cultivated land is expected to be the smallest. The cultivated land is expected to expand rapidly in the economic development scenario, whereas the forest area will be shrinking and the grassland will undergo the most significant drop, which is primarily manifested in the southeast of Acheng District, the northwest of Shangzhi City, and the Songhua River basin of Yilan County.

At the class level, between 2010 and 2020, the patch edges of cultivated land, forest, and grassland turned out to be regular, the patch fragmentation of wetland was intensified, and the distribution of wetland and cultivated land patches tended to be scattered. In the status quo development scenario, the fragmentation of cultivated land, forest, and grassland is anticipated to increase and disperse progressively in the status quo development scenario, while the largest patch of forest land is anticipated to shrink and the edges of the grassland and water areas are expected to become simpler. In the ecological scenario, the scattered and fragmented state of cultivated land, forest, and grassland has improved compared with that of the status quo scenario, the maximum patch index of forest land is expected to increase, and the overall conditions of water areas are expected to improve. Overall patch fragmentation and edge regularization are expected to be most severe in the economic development scenario.

At the landscape level, from 2010 through 2020, both SHDI and SHEI have increased, and the overall landscape level tends to be diversified and homogenized. The connectivity between cultivated land and forest decreased. The SHDI and SHEI are predicted to decrease significantly in each of the three future scenarios, with the economic development scenario showing the most significant decline. CONTAG is expected to decrease slightly in the status quo scenario and is expected to increase slightly in the ecological scenario and the economic scenario, and the relative increase is relatively large in the economic scenario. The 2030 divisions all show small declines, with the largest decline in the economic scenario.

Among the wide variety of development scenarios, the sustainable development scenario has the most significant ecological and economic benefits, and the total benefit is anticipated to be CNY 435,860.88 million. According to this scenario, the study area should have 22,299.88, 19,379.10, 4271.00, 920.74, 4648.54, 2453.68, and 17.24 km^2^ of cultivable land, forest, grassland, wetland, water, artificial surface, and bare land in 2030, respectively.

## Figures and Tables

**Table 1:** The spatial driving factors of the land cover change in this research.

Category	Data	Data Resource
Land Cover	Land Cover Data (2010, 2020)	National Catalogue Service for Geographic Information (http://www.globallandcover.com/ accessed on 1 January 2023)
Natural	DEM	Geospatial Data Cloud (www.gscloud.cn accessed on 1 January 2023)
	Slope	Calculated based on DEM data
	Aspect	Calculated based on DEM data
	Annual Mean Temperature	High-resolution gridded datasets (https://crudata.uea.ac.uk/cru/data/hrg/ accessed on 1 January 2023)
	Annual Mean Precipitation	High-resolution gridded datasets (https://crudata.uea.ac.uk/cru/data/hrg/ accessed on 1 January 2023)
Social	Population Density	World Pop (https://www.worldpop.org/ accessed on 1 January 2023)
	GDP	Geographical Information Monitoring Cloud Platform (http://www.dsac.cn/ accessed on 1 January 2023)
Traffic	Distance to River	Calculated based on DEM data
	Distance to National Highway	National Catalogue Service for Geographic Information (https://www.webmap.cn/ accessed on 1 January 2023)
	Distance to Highway	
	Distance to High-Speed Road	
	Distance to County Road	
	Distance to Railway	
Limiting factor	Natural Reserve	Resource and Environment Science and Data Center (https://www.resdc.cn/ accessed on 1 January 2023)

**Table 2:** Landscape index selection and ecological significance.

Index	Computational Formula	Ecological Significance
NP	NP=N	It represents the total number of all patches, and the greater the number, the greater the degree of landscape fragmentation and landscape spatial heterogeneity.
ED	$ED=\sum_{i=1}^{n}e_{i}$	It is used to demonstrate the degree to which boundaries fragment a landscape or type; the greater the density of boundaries, the more clearly the landscape is broken.
LPI	$LPI=\frac{Max\left(a_{ij}\right)}{A}\left(100\right)$	It shows what percentage of the largest patch of the landscape makes up the overall region, aids in identifying the dominant type of landscape, and can show the direction and level of human activity.
AI	AI=∑i=1n(gimax→gi×pi]×100%	It shows the level of aggregation between landscape patches; the higher the level of aggregation between similar patches, the greater the level of aggregation among landscape patches.
SHDI	$SHDI=-\sum_{i=1}^{m}[p_{i}\ln(p_{i})]$	The comprehensive characterization of landscape richness and complexity indicates landscape heterogeneity. The richer the land use is, the higher the degree of fragmentation is and the higher the SHDI value is.
SHEI	$SHEI=-\sum_{i=1}^{m}\left[p_{i}\ln(p_{i}\right)]/\ln m$	It is used to measure the uniformity of landscape patch distribution in the area.
CONTAG	$CONTAG=\left[1+\frac{\sum_{i=1}^{m}\sum_{j=n}^{m}p_{\left(if\right)}\ln p_{\left(if\right)}}{2\ln m}\right]\times 100$	The degree of aggregation or spread of different patch types in a landscape. A dominant patch type in the landscape creates a good connectedness when the values are high.
DIVISION	*DIVISION* = *D_ij_/A_ij_*	The landscape segmentation index indicates the separation degree of patches in the landscape, and the larger the value, the more fragmented the patch composition and the more complex the landscape.

**Table 3:** Constraint conditions of the objective function of the multi-objective programming model.

Constraint Factors	Constraint Condition/km²	Constraint Explanation
Cultivated land area	*x*_1_ ≥ 22,299.88	To ensure food production and meet people’s dietary needs, cultivated land area is required to be no less than 85% of the 2020 level
Forest area	*x*_2_ ≥ 19,379.10	As a source of ecological services, forests can effectively prevent soil erosion, and the area of forest land should not be lower than the 2020 level
Grassland area	*x*_3_ ≥ 4,271.00	Grassland can improve soil, prevent wind, fix sand, and beautify the environment, and the area of grassland should not be lower than the 2020 level
Wetland area	*x*_4_ ≥ 920.74	Wetlands are the “lungs” of Harbin and can effectively regulate groundwater. The area of wetlands should not be lower than the 2020 level
Water area	*x*_5_ ≥ 842.63	The water area not only is an indispensable part of the ecology but also promotes and sustains the development of the local tourism economy. The water area should account for at least 90% of the 2020 water area
Artificial surface area	2453.68 ≥ *x*_6_ ≥ 2230.62	To ensure the sustainable and stable economic development of the study area, the artificial surface area should not be lower than the 2020 level, and the maximum scale increase should not exceed 10% of the 2020 level
Bare land area	17.31 ≥ *x*_7_ ≥ 17.24	Bare land accounts for a very small part. The forecast result of the FLUS model indicates that the bare land area in 2030 is 17.24 km^2^; the bare land should not be lower than the level in 2020 and should not exceed the predicted value in 2030
Total land area	*x*_1_ + *x*_2_ + *x*_3_ + *x*_4_ + *x*_5_ + *x*_6_ + *x*_7_ = 53,990.18	All types of land cover should be converted into each other, and the total land cover should not be changed
Model constraint	*x_i_* > 0, *i* = 1, 2, 3, 4, 5, 6, 7	The decision variables are all non-negative

**Table 4:** Class-level indices.

Metric	Green Space Types	2010	2020	Status Quo Development Scenario	Ecological Protection Scenario	Economic Development Scenario
NP	Cultivated land	4009	3838	3997	3853	3866
	Forest	17,534	15,098	16,353	15,153	15,059
	Grassland	53,422	50,540	50,565	50,558	50,589
	Wetland	243	511	511	511	511
	Water	3660	3086	3071	3087	3071
ED	Cultivated land	11.69	11.50	11.80	11.50	11.62
	Forest	13.77	13.19	13.38	13.20	13.14
	Grassland	13.92	13.12	13.10	13.11	13.10
	Wetland	0.40	0.56	0.56	0.56	0.56
	Water	1.17	1.22	1.22	1.22	1.22
LPI/%	Cultivated land	28.55	27.01	27.14	27.02	27.30
	Forest	9.73	10.31	10.30	10.32	10.26
	Grassland	0.26	0.27	0.27	0.27	0.27
	Wetland	0.32	0.329	0.32	0.32	0.32
	Water	0.60	1.02	1.02	1.02	1.02
AI/%	Cultivated land	94.24	94.08	93.94	94.08	94.06
	Forest	90.15	90.74	90.57	90.73	90.68
	Grassland	58.31	58.53	58.49	58.51	58.48
	Wetland	93.15	91.81	91.81	91.81	91.81
	Water	77.04	81.94	81.96	81.94	81.96

**Table 5:** Landscape-level indices.

	SHDI	SHEI	CONTAG/%	DIVISION/%
2010	1.0371	0.6444	56.7861	0.8649
2020	1.0624	0.6601	56.4332	0.8706
Status quo development scenario	1.0616	0.6596	56.3034	0.8700
Ecological protection scenario	1.0622	0.6600	56.4361	0.8705
Economic development scenario	1.0608	0.6591	56.4549	0.8692

**Table 6:** Land cover type areas in the sustainable development scenario compared to those in 2020.

Land Cover Type Area/km²	Land Cover Types						
	Green Space					Non-Green Space	
	Cultivated Land	Forest	Grassland	Wetland	Water Area	Artificial Land	Bare Land
Sustainable development scenario	22,299.88	19,379.10	4271.00	920.74	4648.54	2453.68	17.24
2020	26,235.15	19,379.10	4271.00	920.74	936.26	2230.62	17.31
Difference value	3935.27	0	0	0	3712.28	223.06	0.07

**Table 7:** Ecological benefit, economic benefit, and comprehensive benefit under different situations.

Scenario Type	Benefit Type/CNY 1 Million		
	Ecological Benefit	Economic Benefit	Comprehensive Benefit
Status quo development scenario	142,446.47	219,210.89	361,657.36
Ecological protection scenario	142,776.51	219,114.55	361,891.06
Economic development scenario	142,044.99	219,676.57	361,721.56
Sustainable development scenario	194,731.82	241,129.06	435,860.88

## Data Availability

Not applicable.
